# More than a symptom: qualitative exploration of embodied control and restlessness in compulsive movement in eating disorders

**DOI:** 10.1186/s40337-025-01423-7

**Published:** 2025-10-24

**Authors:** Paolo Meneguzzo, Elisa Bonello, Patrizia Todisco

**Affiliations:** 1 Department of Neuroscience, University of Padova, Via Giustiniani 2, Padova, 35128 Italy; 2 Padova Neuroscience Center, University of Padova, Padova, Italy; 3 Eating Disorder Unit, Villa Margherita-KOS Group, Arcugnano, Vicenza, Italy; 4 Psycho-nutritional Center, Vicenza, Verona, Italy

**Keywords:** Eating disorders, Compulsive exercise, Qualitative research, Inpatient treatment, Embodiment, Thematic analysis, Narrative change

## Abstract

**Background:**

Compulsive physical activity is a common but underexplored feature of eating disorders (ED). Beyond calorie expenditure, it often serves complex psychological, symbolic, and embodied functions. Understanding how these behaviors are experienced and change during treatment can guide more effective interventions. This study explored the lived experience of compulsive movement in individuals with anorexia nervosa (AN), bulimia nervosa (BN) and binge eating disorder (BED), and examined whether diagnosis or duration influenced narrative change during inpatient care.

**Methods:**

Sixty-five inpatients with EDs (mean age = 22.15 years; range 16–33) completed an open-ended questionnaire within the first week of admission (T0) and during the final week of hospitalization (T1). The Clinical Interview for Compulsive Exercise [10] was adapted to a written format to elicit spontaneous narratives about movement. Reflexive thematic analysis identified shared themes in T0. For the longitudinal analysis, the T0 and T1 narratives were compared between individuals to capture changes in meaning, content, and emotional tone. The original interview domains were merged into five thematic categories to describe improvement, persistence, or worsening, and subgroup comparisons were made by diagnosis and duration (≤ 3 vs. >3 years).

**Results:**

Five overarching themes emerged at T0: control and compensation, emotional regulation, rigidity and rituality, motor restlessness and bodily discomfort, and covert activity/non-exercise movement. By T1, most of the participants described reduced guilt, greater flexibility, and increased self-awareness. However, persistent restlessness and subtle compensatory activity were reported, particularly in the long-duration group (> 3 years). Diagnostic subgroups differed in emphasis: AN participants often framed movement as a moral duty, BN participants as a means of regulating mood, and BED participants in relation to body image concerns or “getting back on track” with healthy routines.

**Conclusions:**

Compulsive movement in EDs is a multifaceted, transdiagnostic phenomenon. Inpatient care can foster meaningful narrative change, although embodied restlessness may require longer-term treatment. Clinicians should address both the behavioral and symbolic dimensions of movement to support long-lasting recovery.

## Plain language summary

Many people with eating disorders (ED) feel a strong urge to move, exercise or stay active, even when it harms their health. This can include formal exercise, constant fidgeting, standing for long periods, or other invisible activity. These behaviors can be about more than burning calories—they can help manage emotions, give a sense of control, or feel like a moral obligation. In this study, we spoke to 65 people hospitalized for anorexia nervosa, bulimia nervosa, or binge eating disorder. During their first week in the hospital and again before they went home, they answered open questions about their experiences with movement. We looked for common themes in their responses and then compared how their stories changed over time. We also explored whether their experiences differed by diagnosis or by how long they had been unwell. We found that most people reported positive changes at the end of treatment. Many described feeling less guilty if they rested, being more flexible in their routines, and having more insight into their behaviors. However, some still felt restless or found it hard to stop subtle forms of activity. People with shorter illness histories tended to be more open to change, while those with longer illness histories more often saw movement as part of their identity. These results show that inpatient treatment can help people change their relationship with movement, but long-standing habits may need more time and different approaches to change. Treatments should address not only physical behaviors but also the personal meanings and feelings related to movement.

## Introduction

Disturbances in physical activity are a long-recognized but still insufficiently understood phenomenon in eating disorders (EDs). Individuals with EDs often display elevated or dysregulated movement patterns that range from overt exercise to more subtle forms of motor restlessness. These behaviors are not confined to weight control or calorie expenditure; they are intertwined with affect regulation, control, and embodied distress. Within this spectrum, it is important to distinguish between hyperactivity—manifesting as restless, impulsive, or automatic increases in movement—and compulsive exercise, which refers to rigid, rule-bound, and internally driven activity aimed at reducing distress or preventing feared consequences, despite physical or psychological impairment [10]. Although traditionally associated with anorexia nervosa (AN), elevated and dysregulated activity patterns have also been observed in bulimia nervosa (BN) and binge eating disorder (BED), indicating that disturbances in movement and activity can represent a transdiagnostic feature rather than a disorder-specific symptom [1, 3, 10, 23, 28]. This conceptual distinction clarifies that compulsive activity differs both from hyperactivity and from normative or health-promoting exercise, highlighting its close link with emotional regulation, control, and embodied discomfort. Patients often engage in structured or covert forms of movement, such as walking, standing, fidgeting, or exercising, which may serve functions beyond calorie expenditure, including mood modulation, emotional numbness, or a sense of bodily control [3, 25].

The complexity of these behaviors is further compounded by their significance based on morality and identity. For many people with EDs, movement becomes a ritualized, rule-bound activity tied to personal worth and self-regulation [7, 10, 16]. It is often described not merely as a behavior, but as a necessity, intertwined with guilt, anxiety, and embodied discomfort when restricted. While quantitative studies have documented the prevalence and correlates of compulsive exercise, few have explored the subjective experience of movement, particularly throughout the course of intensive treatment [11, 12].

Hospitalization presents a critical context in which movement-related behaviors among individuals with EDs are both limited and therapeutically addressed. Inpatient settings often regulate physical activity to promote weight restoration and reduce the risk of compulsive behaviors, which are commonly associated with poorer outcomes and the risk of relapse [4, 35]. While such restrictions may promote insight and behavioral change in some individuals, they can also intensify psychological distress or resistance, especially in patients with established compulsive exercise patterns [9, 13, 20]. Despite growing awareness of the role of movement in the maintenance and treatment of EDs, little is known about how patients themselves construct narratives around movement across the treatment trajectory. Understanding these narratives is clinically relevant because they illuminate the subjective meanings that patients attribute to movement, which may persist beyond symptom remission [30, 36]. Such insights can guide more tailored interventions: for example, by distinguishing between patients who frame activity as a moral duty versus those who emphasize mood regulation, clinicians can adapt strategies accordingly [15, 27]. Narratives thus provide a valuable lens for capturing therapeutic change and designing person-centered care.The present study aimed to address this gap by qualitatively analyzing written responses from patients with AN, BN and BED at two timepoints: admission (T0) and discharge (T1) from inpatient care. Using a thematic approach, we sought to identify transdiagnostic themes in the experience of movement-related behaviors in T0, to explore longitudinal changes across treatment, and to examine whether these changes differed based on diagnostic category or duration of the illness. By centering patients’ voices, this study provides a richer understanding of the embodied and psychological meanings of compulsive activity in EDs and informs more nuanced, person-centered treatment approaches.

## Methods

### Participants and procedure

The final sample consisted of 65 patients (age range = 16–33 years; M = 22.15, SD = 4.15) admitted to the specialized ED unit of Villa Margherita – KOS Group, Arcugnano-Vicenza, Italy. All participants met the diagnostic criteria for an ED, established through the Structured Clinical Interview for DSM-5 (SCID-5) conducted by a senior psychiatrist with extensive expertise in the field.

Eligibility criteria were: (1) age between 16 and 50 years; (2) diagnosis of anorexia nervosa (AN), bulimia nervosa (BN) or binge eating disorder (BED); and (3) completion of qualitative evaluations at both admission (T0) and discharge (T1). The exclusion criteria were the presence of psychotic disorders, intellectual disability, or major neurological conditions.

The diagnostic distribution was as follows: 27 participants with AN, 23 with BN and 15 with BED. No participants with a diagnosis of Other Specified Feeding or Eating Disorder (OSFED) or other specific diagnosis were admitted during the recruitment period; therefore, this diagnosis is not represented in the sample. The mean duration of the illness was 3.11 years (range = 1–6 years). Participants were also categorized according to illness duration, with ≤ 3 years considered early-stage and >3 years considered enduring EDs, consistent with the definitions proposed in previous literature [2, 31]. The average duration of hospitalization was approximately 3 months, reflecting the standard length of the rehabilitation program provided by the Italian healthcare system.

All participants underwent a multidisciplinary inpatient program that included medical monitoring, nutritional rehabilitation, and individual and group psychotherapies. Detailed descriptions of the treatment model are available in Todisco et al. [32, 33].

### Interview and data collection

Data were collected at two time points: within the first week of admission (T0) and during the final week of hospitalization (T1). At each timepoint, participants completed a structured open-ended questionnaire designed to elicit detailed narratives of their experiences with physical activity and bodily movement.

To assess compulsive exercise, we use the Clinical Interview for Compulsive Exercise [10], a structured and transdiagnostic instrument that assesses compulsive movement behaviors in individuals with EDs. This interview assesses two core criteria:

(A) A rigid, excessive and internally driven pattern of exercise aimed at reducing distress or preventing feared consequences; and.
(B) Significant physical or psychological impairment resulting from behavior.

The interview also allows classification into three subtypes: vigorous exercise, increased daily movement, and motor restlessness. In this study, the original interview items were adapted into an open-ended written format to facilitate spontaneous and reflective accounts from participants. The questionnaires were completed in writing, with optional clinician support for clarity or literacy needs; however, no guidance was given regarding content to preserve narrative authenticity.

No predefined subtypes of exercise were provided; instead, participants were encouraged to describe their movement-related behaviors freely, guided by the domains outlined in Dittmer et al. [10]. This allowed the collection of narratives reflecting participants’ own conceptualizations of activity.

All responses were anonymized, assigned a unique participant code, and analyzed according to the qualitative framework. Ethical approval was obtained from the local ethics committee and all participants provided written informed consent. For participants under the age of 18 years, written consent was obtained along with informed consent from a parent or legal guardian, in full compliance with national regulations and the Declaration of Helsinki.

### Analytic strategy

The analytical approach involved two interconnected phases: a reflexive thematic analysis of admission narratives (T0) and a qualitative longitudinal comparison of narrative shifts between T0 and discharge (T1).

First, a transdiagnostic reflexive thematic analysis [5] was performed on T0 responses to identify recurrent themes related to compulsive physical activity and movement-related behaviors. The analysis followed a recursive six-phase process: familiarization with the data, generation of initial codes, grouping of codes into candidate themes, iterative review and refinement among participants, definition and naming of overarching thematic domains, and final selection of representative excerpts. Although the structured interview domains of Dittmer et al. [10] provided an initial framework, codes were generated inductively to capture the nuanced and subjective meanings expressed by the participants. The coding was carried out by the first author and reviewed by two independent researchers with expertise in EDs and qualitative methodology to enhance credibility and trustworthiness.

Second, a longitudinal comparative analysis was performed by aligning the individual responses to T0 and T1 and examining changes in content, structure, and emotional tone. To facilitate synthesis, the original interview domains were merged into five broader thematic categories, which allowed identification of patterns of improvement, persistence, or worsening over time. Comparative interpretations were made between diagnostic groups (AN, BN, BED) and by duration (≤ 3 years vs. >3 years), with a focus on quality and nature of change rather than numerical scoring. Representative quotations were selected to illustrate key differences and continuities, and reflexive memos were used throughout to document analytic decisions, support transparency, and ensure interpretive depth.

## Results

The analysis followed a qualitative longitudinal approach, examining the written narratives at admission (T0) and discharge (T1) in seven original interview domains, subsequently merged into five more general thematic areas to capture conceptual overlaps: (1) compulsion, rules, and discomfort if interrupted; (2) investment in time, interference, and over-exercising; (3) formal exercise despite underweight or weakness; (4) increased incidental activity (NEAT); and (5) motor restlessness and inability to remain still. Within each theme, we classified trajectories of change into improvement, persistence, or worsening. As summarized in Table 1, persistence was the most common pattern across domains (63.1–83.1%), improvement was most frequently observed in compulsion and rules (23.1%), and worsening was most evident in incidental activity (20.0%). In all themes, participants described their experiences in concrete behavioral terms intertwined with strong emotional and cognitive components, underscoring the enduring and multifaceted nature of compulsive movement in the context of eating disorders.

**Table 1:** Baseline endorsement (T0) and change from admission to discharge (*n* = 65)

Domain	T0 Endorsed *n* (%)	Improved *n* (%)	Persistent *n* (%)	Worsened *n* (%)
Compulsion / Rules	65 (100%)	15 (23.1%)	41 (63.1%)	8 (12.3%)
Time Investment / Interference	65 (100%)	8 (12.3%)	53 (81.5%)	4 (6.2%)
Formal Exercise	65 (100%)	6 (9.2%)	48 (73.8%)	11 (16.9%)
Incidental Activity	65 (100%)	4 (6.2%)	48 (73.8%)	13 (20.0%)
Motor Restlessness	65 (100%)	4 (6.2%)	54 (83.1%)	7 (10.8%)

Note. Baseline (T0) indicates the number and percentage of participants endorsing each domain at admission. Because the interview guide systematically explored each of the five domains, all participants provided material that could be coded under every theme at baseline (T0). This does not imply equal intensity across domains, but rather reflects the structure of the data collection. Change categories reflect participants’ trajectories from admission to discharge: Improved (reduced or more flexible activity), Persistent (no major change), and Worsened (increased rigidity or distress). Percentages are calculated on the total sample (n = 65).

### Domain 1—compulsion and loss of control

At T0, all participants (65/65, 100%) described an intense, often irresistible urge to engage in physical activity, typically framed as a daily necessity rather than a flexible choice. This compulsion was frequently accompanied by anxiety or discomfort when unable to exercise, and for some, it was embedded in rigid patterns or fixed routines. Activities ranged from multiple daily training sessions to constant walking, often without designated rest days.

By T1, the patterns of change varied. Fifteen participants (23.1%)reported a partial reduction in compulsion, with greater ability to limit or structure activity, although internal pressure often persisted. The majority (41/65, 63.1%) showed little to no change, maintaining the same frequency and rigidity. Eight participants (12.3%) appeared to have developed new or intensified compulsive tendencies during admission.

Improvement: Some participants moved from a position of marked anxiety and strict adherence to activity at T0 toward a slightly more regulated approach at T1, sometimes allowing themselves to reduce activity or break routines.

*‘At T0*,* I had to do a lot of physical activity*,* and if I did less*,* I became anxious. At T1*,* I now do my planned 30 minutes*,* though in my mind I would like to do more.”* [Alice, AN, duration of the illness 4 years].

*“At T0*,* I needed to do physical activity several times a day*,* with only one rest day a week. At T1*,* I still feel the need every day*,* but sometimes I try to remain seated*,* even if I feel guilty. ‘* [Anna, AN, duration of illness 1 year].

Persistence: For many, the level of compulsion and associated discomfort remained unchanged, with physical activity still framed as a daily obligation:

*‘At T0*,* I had to walk or at least avoid sitting still; At T1*,* I still need to walk as much as possible in terms of hours and steps.’* [Alessia, AN, duration of the illness 1 year].

*“At T0*,* I exercised up to four times a day; in T1*,* this remained the same.”* [Giorgia, AN, duration of illness 2 years].

Worsening: A smaller group did not report compulsion at T0, but described emerging or increased urges to be active at T1:

*‘At T0*,* I had no compulsion; At T1*,* I feel uncomfortable if I don’t do physical activity*,* even though I don’t have a fixed routine.”* [Annarosa, AN, duration of the illness 2 years].

*“At T0*,* no; at T1*,* sometimes I need to walk for a fixed time and follow an exercise routine’* [Liliana, AN, duration of the illness 2 years].

These findings suggest that while some patients experienced a qualitative softening of compulsive drives during inpatient treatment, for others, the core urge to move remained resistant to change, and in some cases, new compulsive patterns emerged.

### Domain 2—time investment, interference, and over-exercising

At T0, all participants (65/65, 100%) reported dedicating substantial amounts of time to physical activity, and many describing daily durations that significantly interfered with other aspects of life. For some, this meant multiple hours per day and the sacrifice of social or leisure activities; for others, even shorter exercise sessions were framed as obligatory and emotionally charged. Many recognized that they were exceeding what they considered reasonable levels but were unable to reduce.

By T1, the changes were mixed. Eight participants (12.3%) reported modest reductions in exercise time and greater balance with other activities, while the majority (53/65, 81.5%) maintained or even increased their daily duration. Four participants (6.2%) worsened qualitatively, reporting increased guilt or internal pressure, while for most, the emotional framework of the time spent exercising remained constant.

Improvement: A minority described reducing the total time devoted to physical activity or shifting toward a more flexible approach.

*‘At T0*,* I exercised a lot and saw it as an obligation I couldn’t give up; At T1*,* I do 30–50 minutes and acknowledge that it takes time from other things I enjoy.’* [Alice, AN, duration of the illness 4 years].

*“Before admission I usually exercised for at least one hour; here I exercise for about 30 minutes.’* [Maria, AN, duration of illness 2 years].

Persistence: For others, time investment remained relatively stable with little qualitative change, sometimes with minor increases that did not substantially alter the pattern of behavior.

*‘At T0*,* 30 to ten minutes; at T1*,* about 20 minutes daily.”* [Susanna, AN, duration of the illness 5 years].

Worsening: In some cases, although the amount of exercise did not increase markedly, the psychological experience became more negative, with greater guilt or escalating internal pressure.:

*‘At T0*,* about one hour a day*,* feeling forced; At T1*,* I still think it’s too much and feel compelled to continue increasing the level.’* [Alice, BN, duration of the illness 5 years].

*“Two hours a day at T0 and at T1 still many hours*,* with great guilt and anxiety if I don’t do it.”* [Alessia, AN, duration of the illness 1 year]Overall, while some patients reduced the quantitative load of exercise, the qualitative experience, marked by feelings of obligation, guilt, or a sense of ‘never enough’, often remained unchanged.

### Domain 3—formal exercise despite underweight or weakness

At T0, all participants (65/65, 100%) described engaging in structured intentional forms of exercise—such as cardio workouts, resistance training, or high intensity sessions, despite being underweight, physically weak, or medically advised to limit activity. For some, this training was carried out daily for prolonged periods, often embedded within strict schedules. Activities ranged from multiple daily walks combined with floor exercises, to gym-based cardio, to repeated short bouts spread throughout the day.

At T1, 48/65 (73.8%) continued to perform formal exercise at similar or even greater volumes, with persistence of the belief that such activity was necessary or non-negotiable. Only a small group (6/65, 9.2%) report significant reductions. Eleven participants (16.9%) described an increase in formal exercise after admission, starting from low or no activity.

Improvement: A small number reported some change in format or reduction in total time, but the overall necessity to engage remained:

*‘At T0*,* I walked a lot and did two hours of exercises daily; At T1*,* I still do cardio at the gym every day*,* but I try to limit it to the post-lunch period.’* [Alice, AN, duration of the illness 4 years].

*“At T0*,* daily walks and exercises on the floor for one to two hours; at T1*,* still daily walks and exercises*,* although with some adjustments.”* [Alessia, AN, duration of the illness 1 year].

Persistence: For many, structured exercise continued with little change in frequency or intensity, despite ongoing physical vulnerability.

*‘At T0*,* daily elliptical sessions of 35 minutes; at T1*,* still elliptical or bodyweight exercises every day*,* sometimes increasing the difficulty.’* [Annarosa, AN, duration of the illness 2 years].

*“At T0*,* short daily body weight plus one hour of brisk walking; in T1*,* walking in the ward every day for about 30 hours a week total’.* [Anna, AN, duration of illness 1 year]

Worsening: A minority described an increase in formal exercise after admission, starting from low or no activity.

*‘At T0*,* I did no such exercises; At T1*,* I began doing push-ups*,* squats*,* sit-ups and mountain climbers for 10 minutes every day.’* [Liliana, AN, duration of the illness 2 years].

These narratives suggest that for many patients, formal exercise is a deeply rooted behavior, often persisting or intensifying during inpatient care, even in the presence of significant underweight or physical weakness.

### Domain 4—increased incidental activity (NEAT)

At admission (T0), all participants (65/65, 100%) reported deliberately increasing non-exercise activity: walking instead of taking transport, choosing stairs over lifts, performing household chores more vigorously, or prolonging standing time. These behaviors were often consciously implemented as a means of ‘add’ activity outside of formal exercise, sometimes with specific time goals.

By discharge (T1), 48/65 (73.8%) maintained these behaviors, four participants (6.2%) reduced them, and the majority framed these adjustments as intentional and bound to rules, often monitored in terms of hours per week. Thirteen participants (20.0%) increased their NEAT, often making behaviors more frequent and rule-bound.

Improvement: Reports of reduced incidental activity were rare; In many cases, participants described adjustments but not genuine decreases.

*‘At T0*,* I increased daily movement by approximately an hour without specific rules; At T1*,* I still do this almost every day*,* totaling about 30 hours a week*,* keeping to the schedule established by the disease.’* [Alice, AN, duration of the illness 4 years].

*“At T0*,* I walked or did household chores to avoid sitting; At T1*,* I maintained all these behaviors*,* totaling approximately 28 hours a week.”* [Alessia, AN, duration of the illness 1 year].

Persistence: Many maintained consistent NEAT patterns, sometimes modifying the type of activity but not the intention or total duration.

*‘At T0*,* six days a week for at least 15 hours - gymnastics*,* walks*,* chores; At T1*,* I still do stairs instead of lifts*,* housework*,* arranging clothes*,* adding at least 10 extra hours a week.’* [Anna, AN, duration of the illness 1 year].

*“At T0*,* I increased movement by moving objects room to room and doing chores daily; At T1*,* I now watch TV standing or repeatedly climbing stairs’* [Giorgia, AN, duration of illness 2 years].

Worsening: Some participants who reported relatively moderate incidental activity at T0 described an increase at T1, with behaviors becoming more frequent and rule-bound.

*“At T0*,* I increased about one hour of movement daily without specific rules; at T1*,* I engaged in 30 hours a week*,* with strict rules about when and how to move.”* [Alice, AN, duration of the illness 4 years].

*“At* *T0*, *I normally walked instead of taking transport; at T1*, *I set the rule of at least one hour per day of walking or equivalent activity.”* [Monica, AN, duration of the illness 5 years]

Overall, NEAT-related behaviors appeared resistant to change during hospital care, often persisting as a subtle, yet sustained form of movement driven by the same compulsive tendencies as formal exercise.

### Domain 5—motor restlessness and inability to remain still

At admission (T0), all participants (65/65, 100%) reported difficulty remaining seated or still, often describing a constant need to move legs, change posture or stand up. This restlessness was frequently associated with feelings of anxiety, tension, or guilt, and was described as occurring automatically or outside of conscious control. For some, movements were highly deliberate; for others, they were experienced as involuntary habits.

At discharge (T1), the majority (54/65, 83.1%) continued to experience motor restlessness, with little evidence of sustained reduction. In many cases, restlessness persisted daily, sometimes quantified in terms of hours per week, and was framed as difficult or impossible to control. Four participants (6.2%) reported partial improvement, typically through attempts at conscious regulation, whereas seven participants (10.8%) described an increase in restlessness during admission.

Improvement: Only isolated examples indicated even partial improvement, typically involving attempts to consciously regulate movement rather than elimination of restlessness.

*‘At T0*,* restlessness was constant*,* I had to get up immediately; At T1*,* I still struggle every day*,* but I try to control it more.’* [Alice, AN, duration of the illness 4 years].

*“At T0*,* I could not sit comfortably; At T1*,* I still moved constantly but tried to limit it.’* [Alessia, AN, duration of the illness 1 year].

Persistence: For most, the difficulty of remaining still continued with minimal change:

*‘At T0*,* anxiety*,* tension*,* and guilt manifested whenever I thought of skipping activity; At T1*,* I still move my right leg constantly.’* [Anna, AN, duration of the illness 1 year].

*“At T0*,* I moved my legs whenever seated; at T1*,* I still move them all the time.’* [Giorgia, AN, duration of illness 2 years].

Worsening: A smaller group described an increase in motor restlessness during admission:

*‘At T0*,* no restlessness; At T1*,* I move my leg compulsively for about half an hour daily.’* [Annarosa, AN, duration of the illness 2 years].

*“At T0*,* only occasional unconscious movements; In T1*,* I more often bounce my crossed leg when seated.”* [Angelica, AN, duration of the illness 2 years].

Overall, motor restlessness appeared to be a particularly resistant feature, often persisting despite treatment and, in some cases, emerging or intensifying during admission.

### Differences between diagnoses

Across the five thematic areas, the core features of compulsive movement, perceived obligation, rigid rules, and discomfort or guilt when unable to exercise, were observed in participants with AN, BN, and BED. Table 2 summarizes the qualitative differences between diagnoses.

**Table 2:** Diagnostic patterns in compulsive movement themes

Theme	AN	BN	BED
1. Compulsion, rules, discomfort	Highly prevalent; often embedded in fixed routines with minimal flexibility	Common; often linked to compensatory strategies after eating episodes	Less frequent but present; described more in terms of general activity “drive” than rigid rules
2. Time investment & interference	Long daily durations, prioritized over social/leisure activities	Variable; periods of high time use often tied to mood regulation	Lower average time; interference less commonly noted
3. Formal exercise despite underweight/weakness	Frequent, multi-modal, rule-bound	Present but more focused on cardio for calorie burning	Rare; when present, lower intensity and frequency
4. Increased incidental activity (NEAT)	Deliberately maximized; often quantified in hours/week	Present but less rule-bound; used to offset eating	Occasionally present; framed as “keeping busy” rather than structured
5. Motor restlessness	Very common, often severe, and difficult to control	Common; sometimes framed as agitation or “nervous energy”	Less frequent but described as discomfort in stillness

Compulsion was highly prevalent in AN (23/27; 85.2%), common in BN (17/23; 73.9%), and less frequent in BED (7/15; 46.7%). In AN, these behaviors were often embedded in fixed routines with minimal flexibility. In BN, they were more often linked to compensatory strategies after eating episodes. In BED, compulsive features were reported more in terms of a general “drive” to be active than as rigid rules.

Long daily durations of exercise were characteristic of AN (21/27; 77.8%), while in BN they were variable, often tied to mood regulation (13/23; 56.5%), and in BED were less frequent (5/15; 33.3%). Interference with social or leisure activities was more frequently described in AN and BN, and less commonly in BED.

Engagement in structured, intentional exercise was very frequent in AN (20/27; 74.1%), present but more cardio-focused in BN (12/23; 52.2%), and rare in BED (3/15; 20.0%).

Deliberate efforts to maximize incidental activity were reported in AN (18/27; 66.7%), less frequently in BN (9/23; 39.1%), and only occasionally in BED (4/15; 26.7%).

Difficulty remaining still was described by most participants with AN (22/27; 81.5%), by a smaller majority with BN (14/23; 60.9%), and less frequently in BED (6/15; 40.0%).

In summary, compulsive movement was transdiagnostic, but its prevalence and form varied: AN participants most often described high-volume and rule-bound activity, BN participants emphasized guilt regulation and compensatory motives, and BED participants—though fewer in number—reported compulsive features when present, but typically with lower intensity and less rigid structure. Quantitatively, persistence was the most common trajectory across all diagnoses (ranging from 68% in AN to 80% in BN and 73% in BED), while improvement was somewhat more frequent in AN (up to 26% in compulsion/rules) compared to BN and BED. Worsening was observed in all groups but was relatively more common for incidental activity in BN (≈ 22%) and for formal exercise in AN (≈ 19%).

### Differences by duration of the illness

The qualitative patterns differed significantly between participants with 3 years of illness and those with > 3 years. Of the 65 participants, 32 (49.2%) reported an illness duration < 3 years and 33 (50.8%) > 3 years. These groups showed different trajectories of change. In the shorter-duration group, improvement was observed in 6–9 participants (18–28%) depending on the domain, and persistence was the most frequent pattern (≈ 20–23/32, 62.5–71.9%). These participants more often described experimenting with flexibility and rest, sometimes framing reductions in activity as part of engaging with treatment:

*‘Before coming here*,* I used to exercise at least an hour a day; now I limit myself to about 30 minutes and sometimes skip it altogether.’* [AN, duration of the illness 2 years].

Such changes were frequently narrated with ambivalence: reductions were recognized but paired with lingering urges.

*‘I can sit for a while now*,* but I still think about getting up and walking.’* [BN, duration of the illness 3 years].

On the contrary, participants with > 3 years of illness more often described a stable and established activity pattern from T0 to T1Persistence was particularly common in this group (25–28/33, 75.8–84.8% across domains), whereas improvement was rare (2–3/33, typically below 10%). Worsening was observed in both groups, affecting 3–5 participants in each subgroup (≈ 10–15%). These narratives emphasized predictability and habit, with little sign of flexibility:

*‘I still have to walk every day and for the same number of steps; if I reduce*,* the anxiety is too much.’* [AN, duration of the illness 7 years].

‘I *have been doing chores standing up for years*,* and nothing has changed here; I still do them all.”* [BN, duration of the illness 8 years].

In motor restlessness *and* increased incidental activity, longer-duration cases often portrayed these behaviors as automatic and identity-linked, making change seem implausible:

*‘At T0 and T1*,* I was always on my feet*,* moving clothes from room to another; I can’t sit still. It’s part of me now.’* [AN, duration of the illness 12 years].

Worsening patterns, such as adopting new structured exercise, occurred in both groups, but in shorter-duration cases, this was sometimes framed as a novel coping mechanism within the constraints of inpatient life:

*‘I wasn’t doing structured workouts before*,* but here I started daily push-ups and squats to stay active.’* [AN, duration of the illness 2 years].

These narratives suggest that the duration of the illness shapes not only the likelihood of behavioral change, but also the meaning ascribed to movement. Shorter-duration cases more often saw reduction or modification as a tentative therapeutic gain, whereas longer-duration cases tended to frame movement as a stable, defining element of daily life, resistant to change, and often detached from conscious choice.

## Discussion

### Narrative and symbolic meanings of compulsive movement

This study explored the lived experience of physical activity and movement-related behaviors in people hospitalized for EDs, using a qualitative longitudinal approach. By merging the interview domains into five broader themes: compulsion, rules, and discomfort; time investment and interference; formal exercise despite being underweight; increased incidental activity; and motor restlessness, we identified patterns of improvement, persistence, and occasional worsening across the admission-to-discharge trajectory. Through reflexive thematic analysis at admission (T0), we identified five transdiagnostic themes: control and compensation, emotional regulation, rigidity and rituality, motor restlessness and bodily discomfort, and covert activity and non-exercise movement. These findings echo previous literature highlighting the complex, multifaceted role of compulsive exercise in EDs, often serving as both a behavioral symptom and an emotion regulation strategy [8, 18, 25]. Our findings are grounded in the structured clinical interview proposed by Dittmer et al. [10], which we adopted as the basis for data collection. However, our qualitative approach added depth by revealing moralized meanings, subtle invisible activity, and enduring bodily restlessness that may not be detected in categorical assessments. Specifically, the moral and symbolic meanings attached to movement, the role of physical activity in emotional regulation and embodied control, and the persistence of subtle restlessness even in the absence of structured exercise routines emerged as key experiential dimensions. These findings suggest that the structured clinical interview can be effectively enriched by qualitative narrative inquiry to better explore the subjective significance of compulsive movement. From this perspective, narrative evaluation serves not only as a descriptive tool, but also as a potential therapeutic process in itself, facilitating reflection and change in the way patients relate to their bodies and behaviors.

Consistent with previous studies [1, 21], exercise was frequently described as an obligatory and morally charged behavior, particularly among patients with AN. The strong association between movement and control has been widely documented in AN, where exercise often becomes a central characteristic of the identity and a means to assert autonomy and suppress internal chaos [3, 27, 34]. Our results extend these insights beyond AN, showing that participants with BN and BED also engaged in compulsive, emotionally driven movement, often tied to guilt reduction and affect regulation, though with less rigid structuring.

### Differences by diagnosis and illness duration

When comparing diagnoses and illness durations, certain patterns emerged. At discharge (T1), the participants’ narratives revealed signs of meaningful psychological and behavioral change: reductions in routine rigidity, decreased reliance on movement to mitigate guilt, and greater self-reflection. These changes were observed in diagnostic groups, supporting a transdiagnostic interpretation of compulsive movement. However, motor restlessness and the urge to stand or remain active often persisted, suggesting that these features may be partly independent of diagnosis and more deeply rooted in neurobiological or embodied processes [14, 17].

Regarding the duration of the illness, the overall amount of narrative change was similar in those with < 3 years and >3 years of illness. Qualitatively, however, shorter-duration cases more often demonstrated flexibility and openness to reducing activity, while longer-duration participants described identity-based patterns and persistent embodied distress. This supports the view that chronicity shapes the form rather than the possibility of change, particularly for body-based symptoms [2, 24, 29].

### Clinical implications

Our findings highlight the importance of recognizing compulsive movement not only as a symptom to be managed, but as a narrative and embodied coping strategy. The persistence of non-exercise activity and standing behaviors, even when explicit exercise was restricted, resonates with previous ethnographic and self-report studies documenting ‘invisible’ forms of activity in ED patients [10]. Addressing these subtle behaviors requires therapeutic interventions that go beyond behavioral contracts and monitoring, including embodiment-focused therapies, narrative approaches, and emotion regulation training.

The observation that changes occurred in all diagnostic categories supports a transdiagnostic intervention approach, targeting shared mechanisms such as control, guilt, and bodily discomfort. For patients with a shorter duration of illness, interventions may focus on consolidating early gains in flexibility and reflective capacity, while in longer-duration cases, work may need to address the deeper integration of identity with movement and the entrenched belief systems surrounding activity.

Treatment teams may benefit from combining behavioral management strategies (e.g., activity contracts, structured rest periods) with embodiment-focused therapies (e.g., body awareness training, yoga, movement-based mindfulness) to facilitate a shift from compulsive to intentional movement. Finally, the narrative approach used in this study can itself serve as a therapeutic tool, helping patients externalize and reframe their relationship with movement, foster insight, and support long-term recovery.

### Methodological considerations and future directions

Finally, the use of qualitative longitudinal data proved particularly valuable in illuminating subjective shifts that are often overlooked by conventional outcome measures. Standard indices, such as weight restoration or frequency of binge-purge episodes, may not capture the nuanced psychological and experiential changes that patients undergo during treatment. Recent critiques have underscored the limitations of such metrics, highlighting their insufficiency in reflecting internal processes such as motivation, self-reflection, or changes in embodiment [19, 30, 36].

In contrast, narrative data revealed shared experiential processes between diagnoses and durations of the illness, strengthening the relevance of person-centered and transdiagnostic care. Based on these findings, we suggest that future treatment models integrate narrative-based approaches, such as guided autobiographical reflection or therapeutic writing, to help patients externalize and reframe their relationship with movement [15, 27]. Additionally, somatic and sensorimotor therapies (e.g., body awareness training, movement-based mindfulness, or trauma-informed yoga) may be valuable adjuncts to traditional inpatient care, particularly for addressing persistent motor restlessness and nonverbal bodily discomfort [6, 22, 26].

These embodied interventions could support the transformation of movement from a compulsive symptom into an intentional, regulated form of self-expression, ultimately promoting deeper and more sustainable recovery.

Future studies could also explore how these movement-related narratives interact with broader constructs such as shame, identity, trauma, and interoceptive awareness. Comparative work across illness durations and diagnostic categories could clarify whether the qualitative differences we observed, such as greater flexibility in short-duration cases versus identity entanglement in chronic cases—are consistent across larger samples. The development and evaluation of targeted interventions, such as narrative therapy, body-based mindfulness, or sensorimotor psychotherapy, may provide new tools to address the somatic and narrative complexity of compulsive movement in EDs.

### Limitations

Several limitations must be acknowledged. First, the modest sample size limited subgroup comparisons, particularly for the BED and for the > 3-year illness duration, which means that diagnostic and chronicity-related observations should be interpreted with caution. Although qualitative analysis provided rich information, it relied on written responses, which may disadvantage participants with limited verbal expressivity or information. The absence of triangulation with objective behavioral data (e.g., actigraphy, posture monitoring) or psychophysiological measures limited our ability to confirm whether reported reductions in activity were accompanied by measurable behavioral changes. In addition, the analysis focused on subjective reports; triangulation with objective behavioral data (e.g., actigraphy) or psychophysiological measures would strengthen future investigations. Lastly, while our design captured short-term change, post-discharge follow-up is needed to assess whether narrative and experiential shifts, particularly reductions in guilt and rigidity, are sustained over time and whether these shifts predict long-term recovery outcomes.

## Conclusions

This study offers a nuanced understanding of compulsive physical activity in people with EDs by combining thematic and longitudinal qualitative analysis. The findings reveal that beyond diagnostic labels, patients describe movement as a deeply embodied strategy tied to control, emotional regulation, and identity. Our analysis showed that these experiences and the potential for change are shared between AN, BN, and BED, with only qualitative differences in emphasis, and that the duration of the illness shapes the form rather than the possibility of change. Although structured inpatient treatment can foster meaningful narrative and behavioral changes, particularly in reducing guilt and rigidity, some forms of restlessness and embodied discomfort may persist, especially in patients with longer duration of the illness.

These results underscore the importance of viewing compulsive activity not solely as a behavioral symptom, but as an expression of underlying psychological and bodily distress. A person-centered, transdiagnostic approach that incorporates narrative exploration, body-focused interventions, and attention to the duration of the illness may better address the complexity of movement-related experiences in EDs. Ultimately, capturing patient lived experiences across time can offer powerful insights into recovery processes and guide more personalized and compassionate care.

## Data Availability

Due to the sensitive and potentially identifiable nature of qualitative interview data, full transcripts are not publicly available. Deidentified excerpts relevant to the findings are available from the corresponding author upon reasonable request, in line with ethical approvals.
